# Chitosan-Based Oleogels: Emulsion Drying Kinetics and Physical, Rheological, and Textural Characteristics of Olive Oil Oleogels

**DOI:** 10.3390/md22070318

**Published:** 2024-07-17

**Authors:** Mario Lama, Leticia Montes, Daniel Franco, Amaya Franco-Uría, Ramón Moreira

**Affiliations:** Department of Chemical Engineering, Universidade de Santiago de Compostela, rúa Lope Gómez de Marzoa, s/n, 15782 Santiago de Compostela, Spain; marionicolassebastian.lama@rai.usc.gal (M.L.); leticia.montes.martinez@usc.es (L.M.); daniel.franco.ruiz@usc.es (D.F.); amaya.franco@usc.es (A.F.-U.)

**Keywords:** color, marine biopolymer, novel bioproduct, oil binding capacity, oil oxidation, texture, viscoelasticity, water diffusivity

## Abstract

Oleogels are of high interest as promising substitutes for trans fats in foods. An emulsion-templated method was used to trap olive oil in the chitosan crosslinked with vanillin matrix. Oil in water emulsions (50:50 *w*/*w*) with different chitosan content (0.7 and 0.8% *w*/*w*) with a constant vanillin/chitosan ratio (1.3) were air-dried at different temperatures (50, 60, 70, and 80 °C) and freeze-dried (−26 °C and 0.1 mbar) to produce oleogels. Only falling rate periods were determined during air-drying kinetics and were successfully modeled with empirical and diffusional models. At a drying temperature of 70 °C, the drying kinetics were the fastest. The viscoelasticity of oleogels showed that the elastic modulus significantly increased after drying at 60 and 70 °C, and those dried at 50 °C and freeze-dried were weaker. All oleogels showed high oil binding capacity (>91%), but the highest values (>97%) were obtained in oleogels with a threshold elastic modulus (50,000 Pa). The oleogels’ color depended on the drying temperature and chitosan content (independent of the drying method). Significant differences were observed between air-dried and freeze-dried oleogels with respect to oxidative stability. Oxidation increased with the air-drying time regardless of chitosan content. The found results indicated that drying conditions must be carefully selected to produce oleogels with specific features.

## 1. Introduction

The improvement of population health through diet has been a matter of concern in the last decades. One of the main objectives of this topic is related to the decrease in certain solid fats used in the food industry. These solid fats are highly employed since they provide texture and stability, among other properties, to a wide range of foodstuffs [1]. However, they are also rich in trans and/or saturated fatty acids, causing their regular ingestion to result in well-known adverse health effects. Therefore, the alternative of replacing solid fats with healthier vegetable oils appeared as an obvious solution to administrations and the scientific community [2]. But before liquid oils can be employed in a food-chain process, a solid-like texture must be conferred to them. This can be achieved by the oleogelation process [3], which is being extensively studied in recent years. Different methods (indirect and direct), oleogelators (fatty acids, natural waxes, ethylcellulose, alginate, xanthan gum, gelatin, among others), and oils (canola, sunflower, flaxseed, olive, etc.) have been tested in the related literature, resulting in stable oleogels that can give positive properties to different foods [4].

The production of oleogels by direct methods is in general a simple process, but usually involves high temperatures that can damage, by oxidation processes, the benefits of structured unsaturated oils [5]. However, oxidation values are not usually provided in studies related to oleogel synthesis [6,7,8,9]. Therefore, this methodology must be especially reviewed, at least when the final use is going to be the food industry. Indirect methods consist of the preparation of an oil-in-water (O/W) emulsion using milder temperatures, but also include a subsequent dehydration process to obtain the oleogel [10,11,12]. Hence, both emulsion preparation and drying methods can influence the final characteristics of the produced oleogels. Several authors have analyzed the influence of the dehydration method used on the properties of oleogels [13,14,15]. Drying is usually the most energy-consuming operation in an industrial process. Therefore, the optimization of the operational conditions of this stage is important not only to oleogel characteristics but also to the feasible industrial application of oleogels’ production processes. However, scarce studies are focused on analyzing this issue [16]. 

An oleogelator can be defined as a substance added in small proportion to structure a liquid oil in the form of a gelled-like solid. A wide variety of polysaccharides of different origins and properties have been applied in the related literature. A tendency towards employing natural, functional, and sustainable structuring agents can be observed in recent works, like natural triterpene saponin [14], hemp seed protein [17], potato starch [18], or brewery industry by-products [19]. Chitosan is a biopolymer with beneficial health properties that comes from the deacetylation of chitin, a very abundant natural polymer like cellulose [20]. It is found in the structures of marine animals like crustaceans and in insects. Therefore, the interest of chitosan relies not only on its health properties but also on its sustainability, since it can be obtained from abundant marine subproducts of the fish processing industry traditionally considered as an organic waste, like, for example, crustacean shells [21] or squid pens [22]. Thus, the extraction of this bioactive compound either from marine subproducts or other non-edible marine sources (bycatch) can improve the bioeconomy of marine value chains and preserve marine ecosystems [23]. Chitosan is a promising oleogelator, being successfully employed in the production of oelogels [8,9,24], but its low solubility in oil obliges to employ the indirect method to produce oleogels. Specifically, it has been shown that the reactivity of the amino and hydroxyl groups of chitosan is high and can be modified to form Schiff bases [25] with crosslinking agents such as vanillin, glutaraldehyde, or glyoxal [26,27]. Based on these properties, Brito, Di Sarli Peixoto, Martins, Rosário, Ract, Conte-Júnior, and Torres [24] elaborated and improved the stability of the oil-water emulsion of chitosan, adding vanillin to the mixture. These authors, in agreement with others [26], proved that the amino groups from chitosan reacted with the aldehyde groups of vanillin to form the Schiff base bond, along with the hydroxyl group of vanillin, linking hydrogen bonds with the amino or hydroxyl groups with other chitosan molecules. Among the oils that can be employed, olive oil is an interesting option since it presents a good PUFA profile with proven health benefits and it is the main oil produced in Southern Europe [28].

The objective of this work was to study the influence of the drying method (hot air-drying and freeze-drying), drying conditions (different air-drying temperatures), and oleogelator concentration on the physical, rheological, and textural properties, as well as the oxidative level of chitosan-based oleogels with olive oil. 

## 2. Results and Discussion

### 2.1. Drying Kinetics

**Figure 1:**
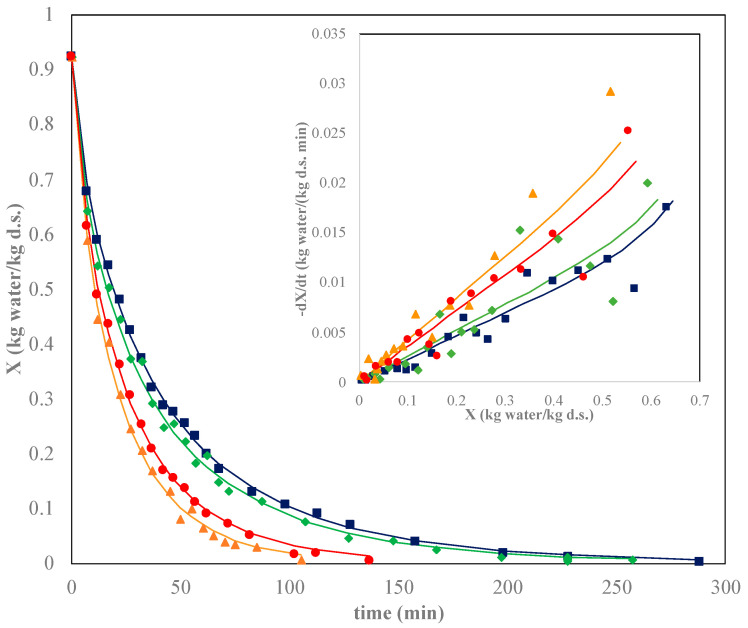
Drying kinetics (main plot) and specific drying rates (subplot) at different air temperatures (°C): 50 ■, 60 ♦, 70 ▲, 80 ● for 0.8% *w*/*w* chitosan content. Lines correspond to the Page model (main plot) and the diffusional model (subplot).

Figure 1 shows the air-drying kinetics and the corresponding specific drying rate curves for the O/W emulsions of 0.8% of chitosan at different air temperatures (data for 0.7% are presented in the Supplementary Material (SM)). For both concentrations, the moisture content initially, during the induction period (<5 min), decreased at a high rate due to the rapid evaporation of the free water present on the oleogel surface. The surface water removal promoted the formation of a crust or gelled soil, which is a common phenomenon in drying known as superficial hardening, that hinders water transport in the bulk of the sample and hence decreases the water removal rate [16]. During drying, the thickness of this layer increased and consequently also the resistance to mass transfer. 

In general, as expected, the drying rate was slower as the drying air temperature decreased, in accordance with the results reported in many references on the drying of different products [29,30]. When higher air temperatures are employed, the solid temperature is also higher, promoting water evaporation from its surface and increasing the internal water diffusivity. Nevertheless, a detailed analysis of drying kinetics indicated a moderate increase in the drying rates of oleogels when the air temperature increased from 50 to 60 °C, but it was pronounced when the air temperature increased from 60 to 70 °C (i.e., drying times shortened from 162 to 70 min and from 227 to 105 min for 0.7 and 0.8% chitosan oleogels, respectively). Increasing the air temperature from 70 to 80 °C did not shorten the drying time. This behavior can be related to the formation of the surface layer/crust, which avoided direct contact between air and emulsion and hindered water transport through the sample. As the drying temperature increased, the formation of this layer was faster, and it could be experimentally observed at the first instants of the drying experiments. The results obtained from the drying kinetics at 70 and 80 °C could indicate that the superficial hardening was very fast above 70 °C and the use of higher air temperatures (i.e., 80 °C) slowed down the drying rate. Hence, from the economical (energy consumption) point of view, 70 °C seemed to be the optimal drying temperature. 

Regarding chitosan concentration, drying times at the same temperature were longer for emulsions with the highest chitosan concentration (0.8%) at a constant drying temperature. Chitosan (with vanillin) was added to immobilize the oil-water system by generating a tridimensional network trapping both oil and water [9,24]. Therefore, a higher chitosan concentration meant greater resistance to moisture removal from the emulsion and a lengthening of drying times. This concentration effect agreed with the results obtained in a previous work using hydroxypropyl methylcellulose (HPMC) as the oleogelator [16]. 

Specific drying rates vs. absolute moisture content plotting for the studied systems (Figure 1 and Figure S1 subplots) clearly showed the gap between the lowest (50–60 °C) and the highest (70–80 °C) temperatures, especially at a moisture content above 0.2 kg water/kg d.s. It can also be appreciated how the highest drying rates were obtained at 70 °C independently of chitosan content. Again, the combined effect of a high temperature (80 °C) and chitosan concentration (0.8% *w*/*w*) could promote the fast formation of a denser gelled layer on the surface, making difficult water diffusion through a more structured and packed network. A constant rate period was not observed, possibly due to the superficial hardening which avoided water evaporation from the surface. In general, the air-drying kinetics of most food products with a high moisture content usually do not present a constant rate period due to free water not being present or the water inside tissue and cellular structures and biological membranes avoiding the direct water-air contact [31]. Specific drying rates (kg water/(kg d.s. min)) decreased (initial moisture content to 0.2 kg water/kg d.s.) from 0.048 to 0.022 at 70 °C, and from 0.035 to 0.006 at 50 °C in the case of emulsions prepared with 0.8% chitosan.

### 2.2. Drying Modeling

Table 1 shows the fitting parameters values obtained by Ms Excel 2016 (Solver add-on) of empirical tested models obtained by nonlinear regression and the corresponding statistical analysis. 

The models applied were the Newton model (NM), the Henderson–Pabis model (HPM), and the Page model (PM), which are described by Equations (2)–(4), respectively. At the same conditions (chitosan concentration and drying temperature), the values of the drying rate constant (*k*, min^−1^, min^−n^) were very similar for the NM and HPM models, and slightly different (although within the same range of magnitude) for the PM model. 

The model parameters increased significantly (*p* < 0.05) with the drying temperature in the range from 50 to 70 °C, and at a high temperature (80 °C) decreased according to the previously described trends of the experimental drying kinetics. At a constant temperature, the value of *k* decreased with increasing chitosan concentration for the NM and HPM, while it increased for the PM. In the Page model, the exponential parameter *n* varied with the drying temperature in the same manner independently of the drying temperature. It was reported that a decrease in this parameter can be related with the product microstructure and varied in a narrow interval at a constant temperature [32], giving as a result longer drying times. In Table 1, *n* presented lower values at high chitosan content of emulsions, which agreed with the experimental results, and increased significantly (*p* < 0.05) with the temperature at a constant chitosan content (0.79 to 0.91 at 0.7% *w*/*w*; 0.74 to 0.83 at 0.8% *w*/*w*). 

Although the three models presented an adequate fitting (*R^2^* > 0.98), the Page model provided the best fitting to experimental results. With this model, the average values of the *φ* parameter were the highest ones (>200), while the *SSE* presented the lowest values (<0.00019). Several works comparing different models selected the Page model as the more adequate to predict the drying kinetics of organic products [33,34,35]. The goodness of the Page model fittings can be observed in Figure 1 and Figure S1, respectively.

The specific drying rate curves of all tested emulsions showed that only the falling rate periods were determined. At these conditions, it was considered that the drying rate was controlled by water diffusion. The effective diffusivity of water during emulsion drying was determined according to Fick’s law. According to the criteria of Crank [36], only the initial drying period (<7 min) can be modeled considering short times (Equation (5)). However, the first minutes of the drying process corresponded to the highly non-steady induction period in which the samples are heated from room temperature up to the conditions given by the drying air employed. Consequently, other coupled phenomena take place simultaneously to water removal, and water diffusivity is strictly hard to be evaluated. Therefore, these initial data were excluded for the water diffusivity determination and only Equation (6) (long times) was applied. The thickness of the sample, the characteristic dimension, was measured during the drying process to determine the shrinkage (Figure S2). An empirical relationship (Equation (S1)) could be established between the thickness and moisture content of samples.

Non-linear optimization with the solver tool (Ms Excel) was employed to estimate effective water diffusivity during emulsion drying considering shrinkage. The goodness of diffusional model fitting for the system at 0.8% *w*/*w* chitosan at different temperatures can be observed in Figure 1 (and in Figure S1 for the 0.7% *w*/*w* chitosan content). The values of effective water diffusivity, *D_eff_*, are collected in Table 2. Water diffusivities decreased as the chitosan concentration increased, since water transport was hindered by the higher solid content of the samples’ drying rate. 

In addition, water diffusivity increased significantly (*p* < 0.05) with the drying temperature from 50 to 70 °C for both chitosan concentrations, while it remained constant (*p* > 0.05) at 0.7% *w*/*w* chitosan content and slowed down (*p* < 0.05) at 0.8% *w*/*w* chitosan content when the drying temperature increased at 80 °C. Statistical parameters (Table 2) showed acceptable goodness (*R^2^* > 0.98, *φ* > 993, and *SSE* < 0.00079) of the diffusional model to experimental data. 

### 2.3. Color Features

#### 2.3.1. Color during Drying

**Figure 2:**
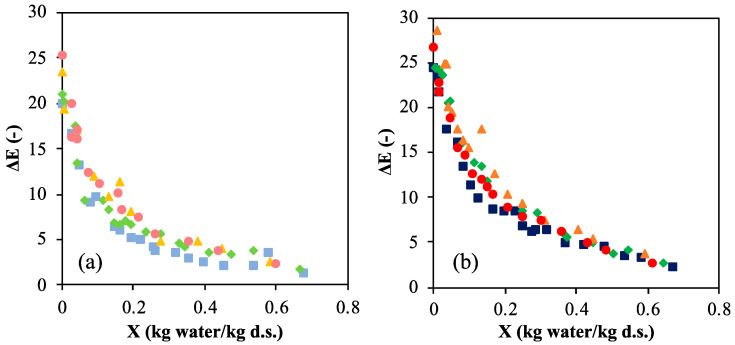
Total color difference, Δ*E*, with moisture content, *X*, of emulsion at different air-drying temperatures (°C): 50 ■, 60 ♦, 70 ▲, 80 ● for chitosan content of (**a**) 0.7% *w*/*w*; (**b**) 0.8% *w*/*w* (darker colors correspond to 0.8% *w*/*w* and clearer ones to 0.7% *w*/*w* chitosan content).

The change in color parameters with moisture content during drying at different temperatures for O/W emulsions with 0.7 and 0.8% *w*/*w* chitosan concentration can be observed in Figure S3 of the SM. The initial values of the coordinates for each system are also shown in the SM (Table S1), employed as references for determining the total color difference during drying. A higher chitosan content led to a slight increase in L* and b* and a decrease in a*. The increase in b*, which indicated yellower tones, can be related to the higher presence of vanillin, which also increased the Schiff bases formed [8]. In general, air-dried samples presented the same behavior. The lightness coordinate (L*) slightly decreased with moisture content until 0.3 kg water/kg d.s., and below this value the decrease in L* was more pronounced. This trend is related to the darkening of the superficial layer due to a possible oil oxidation during drying. At the same temperature, the final value of L* was lower for the most chitosan-concentrated system, since longer drying times were required. Coordinates a* and b* followed the same trend, increasing slightly during the first stages of drying and more pronouncedly at a low moisture content. In fact, low L* and b* values together with high values of a* are indicative of brown color [37]. The effect of the drying temperature can be observed especially for coordinate a*, being its values higher for 70 °C (the faster drying temperature) as browning developed. The total color difference increased as the moisture decreased for all the systems, as seen in Figure 2. At the initial times, color variation was almost imperceptible to the human eye, but it turned more noticeable (3.5 < ∆*E* < 5) at an intermediate moisture content with higher Δ*E* values at high drying temperatures. For example, in systems with 0.7% chitosan, Δ*E* was > 3.5 at 50 °C and *X* = 0.27 kg water/kg d.s. while at 60 °C, this value was reached at 0.41 kg water/kg d.s. At a constant drying temperature, Δ*E* was higher in the most chitosan-concentrated emulsions due to the longer drying times required. 

The freeze-dried samples presented a clear and intense yellow color (Figure S4). This result was expected, since freeze-drying minimizes browning reactions [38]. However, as in the convective-dried samples, L* and b* were lower and a* was higher for the most chitosan-concentrated emulsions. On the other side, Δ*E* was almost identical for both systems (19.5 approx.), although lower than for air-dried samples.

#### 2.3.2. Oleogel Color

Color coordinates were measured for both the dried solid and the oleogel (Table 3) to determine how the oleogel formation after homogenization and subsequent refrigeration for 48 h affected these parameters. The oleogel was much darker than the dried solid, and this was reflected in L* values, which decreased dramatically (from 51.01 to 25.87 for 0.8% *w*/*w* chitosan content and dried at 80 °C, for example). Regarding the other coordinates, a* increased and b* presented lower values, indicating more reddish and cold tones in the oleogel. This result could be related to the restructuration of the sample during the homogenization step with the partial release of oil and the excess of vanillin. Vanillin can be exposed to the surrounding air and oxidized, turning into vanillic acid, which presents a brown color. Regarding the freeze-dried oleogel samples, the same previous trend was observed (higher L* and b*, lower a*). 

The color parameters of oleogels were significantly correlated with the drying temperature (*p* < 0.05), increasing L* at low chitosan content and decreasing at high chitosan content with increasing temperature while a* (−0.33 to 2.25) and b* (12.35 to 17.21) varied in a narrow range with the drying temperature. In general, high chitosan content increased the b* of oleogels dried at the same temperature. 

### 2.4. Rheological Properties of Oleogels

**Figure 3:**
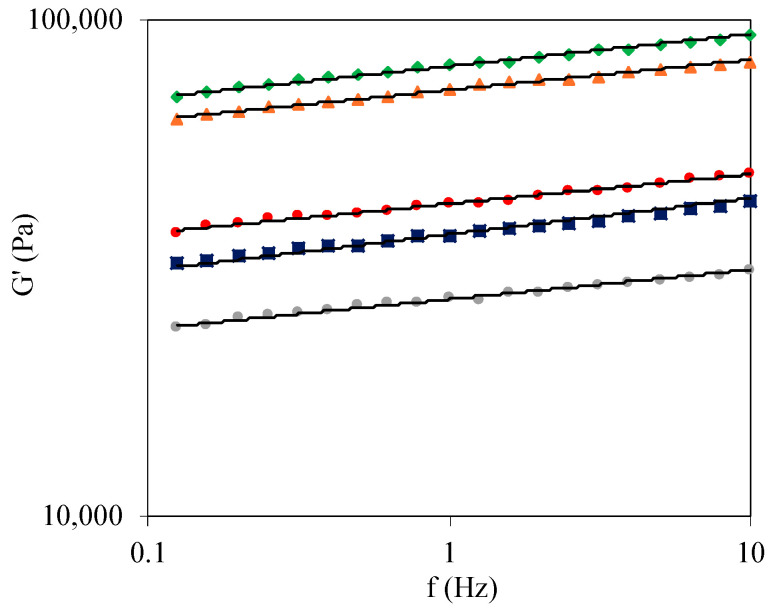
Elastic modulus, *G*′, trend with frequency, *f*, of oleogels formed from emulsion with 0.7% *w*/*w* chitosan concentration, air-dried at several temperatures (°C): 50 ■, 60 ♦, 70 ▲, 80 ●, and freeze-dried: ●.

As expected, the frequency sweeps revealed the predominant elastic behavior of the tested oleogels, with the elastic modulus (*G*′) higher than the viscous modulus (*G*″) in all cases, with a damping factor (tan δ = *G*″/*G*′) less than 1. Specifically, tan δ varied in a narrow interval from 0.10 up to 0.15 in the range of assayed frequency (from 0.1 to 10 Hz) independently of the oleogel chitosan content and drying temperature employed (Figure S5). Figure 3 shows the trend of *G*′ with the frequency of oleogels formed from emulsion with 0.7% *w*/*w* of chitosan content and dried at different temperatures, as an example. In all cases, the elastic modulus increased moderately with the frequency with low slopes (from 0.059 to 0.072), indicating that the gels’ structure was very stable and well formed [39]. Regarding the *G*′ values, it is worthy to note that the highest elastic values were achieved in the oleogels dried at 60 and 70 °C, and the use of lower (50 °C) or higher (80 °C) drying temperatures resulted in weaker gels. Finally, the freeze-dried oleogels showed the weakest structure given by the lowest *G*′ values.

Regarding the oleogels formed with more chitosan concentration (0.8% *w*/*w*), G′ also varied slightly with the frequency (low slopes between 0.062 and 0.077), and the same behavior with temperature was observed, with the highest *G*′ values in oleogels dried at 60 and 70 °C and the lowest values corresponded to freeze-dried oleogels. Table 4 shows the *G*′ values of tested oleogels at the frequency of 1 Hz. These values facilitate the evaluation of the effect of chitosan content on the oleogel strength. A high concentration of the oleogelator increased the strength in oleogels dried at a low temperature (50 °C) and freeze-dried. In the oleogels dried at intermediate temperatures (60 and 70 °C), the opposite behavior was found, with the maximum *G*′ values in oleogels formulated with low chitosan content. Finally, no differences were found in oleogels dried at the highest drying temperature (80 °C) with different chitosan content. These results indicated that at low temperatures (air-dried at 50 °C and freeze-dried oleogels), the use of more oleogelator increased the gel strength as expected, but the gels were weak (*G*′ at 1 Hz < 52,500 Pa). At 60 °C of the drying temperature, the *G*′ values increased (*p* < 0.05) hugely (80,900 Pa) at a low chitosan concentration and more moderately (59,900 Pa) at a high content. This result can be explained by the fact that the increase in temperature decreased the apparent viscosity of the continuous phase (water) and favored the structuration of the chitosan–vanillin matrix, particularly in the case of low concentrated chitosan emulsion, since viscosity increased noticeably with the chitosan content. The oleogels dried at 70 °C showed similar elastic moduli than those dried at 60 °C, but slightly weaker due to, probably, a faster chitosan structuration (shorter drying times). At 80 °C, the superficial hardening, present from the first moments of drying, avoided the proper chitosan structuration, giving as a result the dramatical weakening of final oleogels with low elastic moduli (<44,000 Pa).

### 2.5. Textural Properties of Oleogels

**Figure 4:**
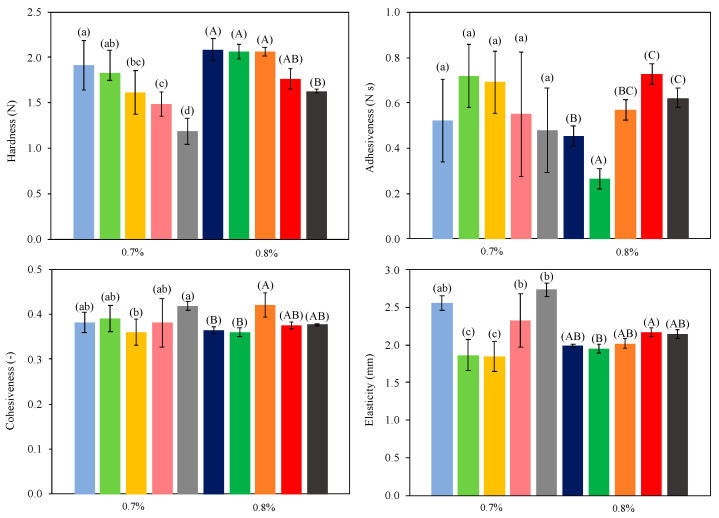
Texture properties of tested oleogels. Dried samples at 50 °C (blue), 60 °C (green), 70 °C (orange), 80 °C (red), and freeze-dried (grey). Darker colors correspond to 0.8% *w*/*w* and clearer ones to 0.7% *w*/*w* chitosan content. Different letters in error bars (a, b, c for 0.7% *w*/*w* chitosan content; A, B, C for 0.8% *w*/*w* chitosan content) indicate significant differences (*p* < 0.05) among mean values at different temperatures according to Duncan’s test.

The results of texture analysis of oleogels can be observed in Figure 4. Hardness, adhesiveness, cohesiveness, and elasticity were measured. The hardness values ranged from 1.19 to 1.83 N and from 1.63 to 2.08 N for 0.7% and 0.8% chitosan concentration, respectively. Harder oleogels were obtained with higher chitosan concentrations. This result was in accordance with other works, where increasing the structuring agent concentration led to an increase in oleogel hardness [10]. Higher drying temperatures reduced oleogel hardness (*p* < 0.05), regardless of chitosan concentration. This result can be explained by the combined effect of longer drying times and milder temperatures, improving the mechanical resistance of oleogels when large deformations are made by compression stress. Although very similar drying times were obtained at 70 and 80 °C, hardness was lower in oleogels dried at 80 °C, meaning that when water diffuses through the rapidly formed structure at this temperature could locally break it. Lower hardness with an increasing temperature and decreasing structuring agent concentration was also obtained in a previous work [16]. Freeze-dried oleogels presented lower hardness (*p* < 0.05) values than convective-dried samples (Figure 4). During freeze-drying, channels and pores can be formed within the solid, leading to a weaker structure [40]. Moreover, the superficial crust formed during convective air-drying can confer additional hardness to the samples. Nevertheless, higher values of hardness were found in freeze-dried oleogels stabilized by gelatin–polyphenol–polysaccharides when compared with air-dried ones [13]. The hardness values were also significantly lower in comparison to oleogels (9.75–20.9 N) structured with chitosan–vanillin and obtained by freeze-drying [24]. These high values can be explained by a different composition of emulsion (40:60 O/W) and the use of a higher concentration of gelling agent. In addition, vanillin was added in a higher amount, resulting in much more structured oleogels [8]. 

Adhesiveness varied in a narrow range (0.45–0.73 Ns) and only increased significantly with increasing drying temperature (*p* < 0.05) at high chitosan content. At low chitosan content, no significant (*p* > 0.05) differences were observed. Adhesiveness increased with chitosan concentration in oleogels dried at 80 °C (and freeze-dried), and the opposite trend was observed in air-dried samples at lower temperatures. Other authors found that adhesiveness and hardness presented similar trends [24]. In this work, in general, as the drying temperature was increased, weaker and stickier oleogels were produced. Cohesiveness varied in a very narrow interval (0.36–0.42), and no clear dependences with the drying temperature and chitosan concentration could be established. Farooq, Ahmad, Zhang, Chen, Zhang [8] obtained similar values (0.35–0.56) in chitosan-based oleogels. 

Elasticity was significantly influenced by both temperature and chitosan concentration. At the lowest, 50 °C (and free-dried samples), and the highest temperature, 80 °C, oleogels’ elasticity increased with decreasing chitosan concentration. The opposite trend was observed at intermediate (60–70 °C) drying temperatures. In general, these results were expected considering hardness values. As stiffness and compaction increase, the shape recovery capacity decreases. Similar results were obtained in other formulations of oleogels [41]. Freeze-dried samples showed high values of elasticity, but not as much as expected considering their low hardness. 

### 2.6. Oil Binding Capacity

Oil binding capacity (*OBC*) is an important quality parameter of oleogels. The *OBC* values are shown in Table 5 for all the tested systems. In general, oil retention values were good (*OBC* > 91%), obtaining the highest values (>97.39%) when air-drying temperatures of 60 and 70 °C (without significant differences, *p* > 0.05) were employed and, specifically, when the highest chitosan content was employed (>98.13%). It was expected that a higher concentration in structuring agent improved oil encapsulation capacity, as observed in previous works [10,15,42]. For instance, Zhu, Liu, Li, Zhang, Wang, Chen, Wang, Xie, Qi, Jiang [12] obtained *OBC* values of 99% in soybean oleogels stabilized with the highest tested (3%) xanthan gum concentration. Farooq, Ahmad, Zhang, Chen, Zhang [8] also found a significant effect of chitosan concentration on *OBC*, while vanillin content seemed to have no influence on this parameter. These authors obtained lower values of oil loss in chitosan–vanillin-based oleogels, but the concentrations employed were higher and the analytical procedure to determine this parameter was milder (less time and rpm) than in the present work. The drying temperature affected significantly (*p* < 0.05) the *OBC*. Oil retention gradually increased from 50 to 70 °C, and decreased at 80 °C to values similar at 50 °C regardless of chitosan content. Freeze-dried oleogels also presented acceptable oil retention (>91.79%), being higher in the 0.8% chitosan samples. However, freeze-dried oleogels showed the lowest values in comparison to the air-dried oleogels. 

**Figure 5:**
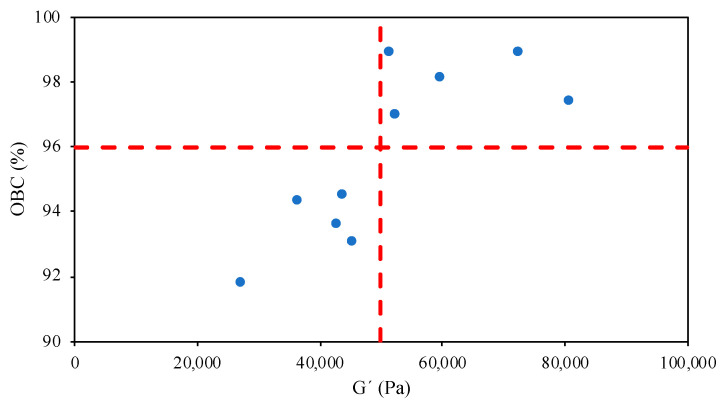
Relationship between elastic modulus, *G*′, and oil binding capacity (*OBC*) of tested oleogels.

As previously mentioned, Zhu, Liu, Li, Zhang, Wang, Chen, Wang, Xie, Qi, Jiang [12] reported high OBC in freeze-dried oleogels, while Abdolmaleki, Alizadeh, Nayebzadeh, Hosseini, Shahin [43] did not find a significant relationship between *OBC* in sunflower oleogels and the drying method (air- and freeze-drying). Miao, McClements, Zhang, Lin, Ji, Wang, Jin, Li, Jiang, Wen, Sang, Qiu [9] obtained values of oil loss between 2 and 14% in oleogels stabilized with octenyl succinic anhydride starch/chitosan. Thus, composition (oil, structuring agent, addition of thickening compounds, and other additional agents) needs to be considered to establish the optimal drying conditions for oleogel production. Furthermore, oleogel strength and *OBC* values were interestingly related (Figure 5). Independently of both the drying temperature and chitosan concentration, *OBC* values above 96% were only obtained in oleogels with *G*′ values >50,000 Pa, meaning there exists a threshold strength of gel able to successfully bind the olive oil in the chitosan structure. 

### 2.7. Oxidative Stability

Oil oxidation is a key parameter for defining oleogel quality. There is no sense in formulating an alternative to trans/saturated fats that do not offer proven health benefits to replacing these products. Or, at least, it is important to ensure that it does not contribute harmful effects. Oxidized oils can contain toxic compounds that negatively affect human health [44].

**Figure 6:**
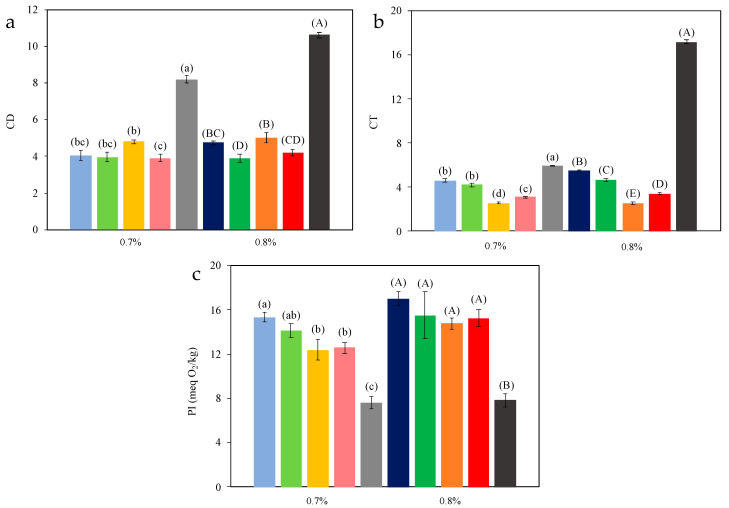
Oxidation parameters for oleogels (conjugated dienes (*CD*, (**a**)) and trienes (*CT*, (**b**)), and peroxide index (*PI*, (**c**))). Dried samples at 50 °C (blue), 60 °C (green), 70 °C (orange), 80 °C (red), and freeze-dried (grey). Darker colors correspond to 0.8% *w*/*w* and clearer ones to 0.7% *w*/*w* chitosan content. Different letters in error bars (a, b, c for 0.7% *w*/*w* chitosan content; A, B, C for 0.8% *w*/*w* chitosan content) indicate significant differences (*p* < 0.05) among mean values at different temperatures according to Duncan’s test.

Figure 6 shows the results for the oleogels’ oxidation. All oxidation methods (conjugated dienes (*CD*), conjugated trienes (*CT*), and peroxides index (*PI*)) provided very similar profiles among the samples. Statistical analysis of the influence of independent variables (temperature and chitosan concentration) on oleogel oxidative stability revealed that drying temperature had a greater effect on the primary oxidation (*p* < 0.001 for *CD*, *CT,* and *PI*). Certainly, a comparison between freeze-drying and convective drying is meaningful, not only in drying temperature. However, the first one is widely used in the oleogel literature; hence, it is presented for comparative purposes. Indeed, freeze-drying typically occurs around −50 °C, whereas the second one operates within the 50−80 °C range. For instance, if freeze-drying is not considered in the statistical analysis, the behavior of the data is the same for the primary oxidation measured by *CD* and *CT*, but not for the *PI* values. In this latter case, the variable chitosan concentration had more influence than the drying temperature during the drying process (i.e., *p* = 0.018 vs. *p* = 0.104 for chitosan concentration and temperature, respectively). The *CD* values were not significantly (*p* > 0.05) affected by the different temperatures tested in convective drying for both chitosan concentrations. However, when freeze-drying was used to obtain the oleogels, the *CD* values increased significantly (*p* < 0.05) from an average value of 4.16 for all temperatures tested in convective drying to 8.18 for chitosan content of 0.7% *w*/*w*. Increasing the chitosan concentration (0.8% *w*/*w*) resulted in *CD* values rising from 4.46 in convective drying to 10.60 (Figure 6a). A similar trend was observed in *CT* results when compared with freeze-drying. On the contrary, when examining the effect of drying temperature within the convective method, a decrease in *CT* values was observed as the temperature increased (Figure 6b). Although this might seem surprising, it is important to note that increasing the drying temperature also reduces the required drying time, significantly impacting oxidation, especially within the moderate temperature range used in this study. Additionally, these results are consistent with the PI values (Figure 6c), showing that increasing the drying temperature and consequently reducing the drying time resulted in a significantly (*p* < 0.05) lower *PI* value (15.32 vs. 12.58 meq O_2_/kg olive oil oleogel for temperatures of 50 °C and 80 °C, respectively).

Unlike for chitosan concentrations of 0.7% *w*/*w*, these significant differences were not found in oleogels at 0.8% *w*/*w* due to the higher variability in the samples indicated by the greater standard deviation (Figure 6b). The peroxide values in lyophilized oleogels significantly decreased (*p* < 0.05) compared to the average of all conventionally dried samples (7.60 vs. 13.61 meq O_2_/kg and 7.82 vs. 15.63 meq O_2_/kg for chitosan content of 0.7% and 0.8% *w*/*w*), meaning a reduction in peroxide values of 44% and 50%, respectively (Figure 6c). This contradiction with the *CD* and *CT* data can be explained by the formation of a porous structure during the freeze-drying process which increases the surface area, oxygen diffusion within the oleogel, and consequently lipid oxidation. These pores can act as diffusion channels for oxygen, oxidizing the sample without browning [45]. Conversely, convective drying of oleogel leads to the formation of a superficial crust and subsequent gelation due to the reaction between chitosan and vanillin, providing superior protection against thermal oxidation. This hypothesis is not supported by *PI* values, but these values could not be representative since the volatile peroxides formed during oxidation could be lost during freeze-drying [46]. 

Finally, focusing on the independent variable of chitosan content (and consequently vanillin) in the stability of the oleogels, some studies have indicated that both chitosan [6,9] and vanillin [47] can act as antioxidants and, consequently, a decrease in oxidation levels would be expected increasing their content, but this potential effect was not found in this study. This fact might indicate that practically all vanillin molecules reacted to form Schiff bases, leaving no free molecules to act as radical scavengers. Another explanation could be related to that vanillin molecules were trapped inside a self-assembled network created with chitosan, preventing its antioxidant action.

## 3. Materials and Methods

### 3.1. Materials

Olive oil (Aceites Abril, S.L., Ourense, Spain) was purchased in a local supermarket, presenting a peroxide concentration <15 meq O_2_/kg and an acidity percentage of 1%. Medium-molecular weight (300.4 ± 18.3 kg/mol) chitosan (Sigma-Aldrich, St. Louis, MO, USA) was employed, being dissolved in a glacial acetic acid (Merck, Darmstad, Germany) solution at 1% *v*/*v*. Vanillin (Sigma-Aldrich) was used in solution (93% purity) with ethanol (Panreac, Barcelona, Spain). 

Isooctane, chloroform, pentahydrate sodium thiosulfate (Panreac), potassium iodide (VWR Chemicals, Leuven, Belgium), and potato starch (Sigma Aldrich) were employed in the experiments related with oil oxidation. 

### 3.2. Preparation of Emulsions

The experimental procedure was based on the work of Brito, Di Sarli Peixoto, Martins, Rosário, Ract, Conte-Júnior, Torres [24], with some modifications. First, 50 g of oil in water emulsions (50% *w*/*w*) was prepared for each experiment, being the aqueous phase constituted by the chitosan and vanillin solution. Chitosan solution was added in each case to achieve a final concentration of 0.7 and 0.8% *w*/*w* of chitosan [24]. A constant vanillin/chitosan ratio (1.3) was kept [24]. Olive oil was added from a burette at a constant flow rate (9 mL/min) on the chitosan solution, which was simultaneously being stirred (150 rpm) by an orbital shaker (Rotaterm, Selecta, Barcelona, Spain), and the mixture was homogenized in a high-energy dispersion unit (Ultraturrax T-25 Basic, IKA-WERK, Staufen, Germany) at 9500 rpm for 3.5 min, followed by the addition of the vanillin solution until completing 4 min of the procedure. After the addition of vanillin, the system was left under homogenization conditions for another 4 min. Afterwards, the emulsion was gently stirred (400 rpm) at room temperature for 2 h, and then it was rested for 24 h, to provide enough time for the reaction between chitosan and vanillin to be completed.

### 3.3. Drying of Emulsion

#### 3.3.1. Convective Drying

Emulsions were placed in 115 mm diameter Petri dishes, which were previously weighted in a precision balance (Mettler PJ3000, Gemini BV, Apeldoorm, The Netherlands). Emulsion was added until reaching a layer of 1.5 mm of thickness. The samples were introduced into a forced convective air-dryer (Challenge 250, Angelantoni, Massa Martana, Italy) for moisture (and ethanol) removal until a dried gelled solid was formed. Emulsions were dried to a moisture lower than 1% *w*/*w*. The drying experiments were performed at four air-drying temperatures (50, 60, 70, and 80 °C), keeping a constant air velocity (2 m/s) and low air relative humidity (10%). 

Drying kinetics (*n* = 3) were obtained by weight monitoring of the samples. The first weight was recorded after 7 min, and then every 5 min during the first hour. As the drying process advanced, measurements were delayed every 15 min and in the last stages, when the drying rates were slower, sample weight was measured every 30 min. Shrinkage was calculated by measuring sample thickness variation during drying with a digital caliper. The drying rates, *-dX/dt* (kg water/(kg d.s. min)), were evaluated and plotted vs. the moisture content, *X* (kg water/kg d.s.), of the gelled-solid sample. Once the convective drying was completed, samples were covered with plastic film and left in darkness for 24 h. 

#### 3.3.2. Freeze-Drying

Emulsion freeze-drying was carried out according to [24]. Once prepared, the emulsion was placed in glass bottles and introduced into a freeze-dryer (LyoQuest-55, Telstar, Barcelona, Spain) at −55 °C for at least 3 h. After, the bottles were stored in a freezer at −26 °C while a vacuum was generated in the freeze-dryer until a pressure of 0.1 mbar was reached, and the sample bottles were carefully placed again in the equipment for 48 h to complete the sublimation step and the drying process. The samples were then stored in a fridge at 4 °C for 24 h more. 

Dried samples (either by air-drying or freeze-drying) were placed in beakers, weighed, and homogenized with Ultraturrax at 9500 rpm. The formed oleogel was stored at 4 °C for 48 h before characterization.

#### 3.3.3. Air-Drying Modeling

Several empirical models can be applied for the simulation of the falling rate period either for deep-bed or thin layer systems. These models usually describe the decrease in the moisture ratio (*MR*) with time. This ratio can be defined by Equation (1):

(1) $$MR=\frac{X-X_{e}}{X_{i}-X_{e}}$$

where *X* (kg water/(kg d.s.) is the moisture content at time *t*, *X_i_* is the initial moisture content, and *X_e_* is the equilibrium moisture content, which is very close to zero for oleogels and can be neglected. Semi-empirical models, like the Newton (or Lewis) model (NM), the Henderson–Pabis model (HPM), and the Page model (PM), are usually employed [30,33], and are defined according to Equations (2)–(4), respectively:

(2) $$MR=e^{-kt}$$

(3) $$MR={ae}^{-kt}$$

(4) $$MR=e^{-kt^{n}}$$

where *k* is the drying constant rate in 1/min for NM and HPM and in 1/min^n^ for PM, *t* is the time (min), and *a* (-) and *n* (-) are model parameters. On the other hand, the inexistence (or the negligible duration) of a constant rate period indicates that internal water diffusion controls the drying rate. Therefore, water transfer can be described by Fick’s law, and the effective water diffusion coefficient at each drying temperature can be determined by the solution proposed by Crank [36], in this case, for a semi-infinite plate. Depending on the drying time, different equations must be applied for short or long times (Equation (5) and Equation (6), respectively):

(5) $$\frac{X_{i}-X}{X_{i}}=1-\sum_{n=0}^{\infty }\frac{8}{(2n+1{)}^{2}\pi^{2}}e^{{-D}_{eff}(2n+1{)}^{2}\frac{\pi^{2}t}{r^{2}}}\;\;\;\forall \left(\frac{X_{i}-X}{X_{i}}\right)<0.4$$

(6) $$\frac{X_{i}-X}{X_{i}}=2{\left(\frac{D_{eff\;}t}{r^{2}}\right)}^{0.5}\left\{\pi^{-0.5}+2\sum_{n=1}^{\infty }(-1{)}^{n}ierfc\frac{nr}{\sqrt{D_{eff}\;t}}\right\}\;\;\;\forall \left(\frac{X_{i}-X}{X_{i}}\right)>0.4$$

where *r* is the thickness of the system (m) and *D_eff_* is the water effective diffusivity (m^2^/s).

The goodness of fit between the experimental and predicted data was defined by *R^2^*, *SSE*, *φ*, the root mean squared error (*RMSE*), and the mean relative deviation (*MRD*), following other works [30,48].

### 3.4. Rheological Characterization

The rheological characterization of oleogels was performed with a stress-controlled rheometer (MCR 301, Anton Paar GmbH, Graz, Austria) with plate–plate geometry (50 mm) and a gap of 1.0 mm at a constant temperature of 25 °C. Firstly, the linear viscoelasticity range (*LVR*) was determined with a strain sweep from 0.01 to 10% at a constant frequency of 1 Hz. After, a frequency sweep from 0.1 to 10 Hz was made at 0.1% of strain to the viscoelastic characteristics of samples. The temperature was controlled by a Peltier system (±0.01 °C). The tests were carried out at least in triplicate.

### 3.5. Textural Properties

Oleogel samples of 19 mm diameter and 8.5 mm height on average were compressed in a texturometer (TA.XT Plus, Stable Micro Systems, Surrey, UK) fitted with a cylindrical probe of 25 mm diameter (SMS P/25). Six sample replicates were analyzed for each oleogel batch. Tests were performed with a 50% compression, being the remaining parameters based on Farooq, Ahmad, Zhang, Chen, Zhang [8]. The initial force applied was 0.1 N, and the speed of the pre-test, test, and post-test in all the experiments was 2.0, 1.0, and 2.0 mm/s, respectively. The maximum hardness force (N) by compression to measure the oleogel firmness, the total necessary work (N m) to compress the oleogel to with an external applied force, and the elasticity modulus (N/m) which related the effort and the deformation were obtained. 

### 3.6. Oil Binding Capacity

The oil binding capacity (*OBC*) of the oleogels was determined according to [49] with minor modifications [16]. The samples (1 g) were introduced in Eppendorf tubes (*n* = 3) previously weighed. The tubes were centrifuged in a minicentrifuge (HW12, HWLAB, Sheridan, WY, USA) at 12,500× *g* for 25 min at 20 °C. Supernatant oil was removed with a Pasteur pipette after centrifugation and the Eppendorf tubes were weighed again. The *OBC* (%) was calculated according to Equation (7):

(7) $$OBC\;\left(\%\right)=\left(\frac{m_{2}-m}{m_{1}-m}\right)\;100$$

where *m* (g) is the weight of the empty Eppendorf tube, and *m*_1_ (g) and *m*_2_ (g) are the weight of the Eppendorf tube with the oleogel sample before and after centrifugation, respectively. 

### 3.7. Oxidation Degree

The determination of the oxidation degree of the oil present in the oleogel implied the extraction of the oil from the oleogel with a syringe with a Luer lock tip and 0.45 micron polypropylene syringe filter. Primary oxidation was quantified by the determination of the peroxides index (*PI*) and conjugated dienes (*CD*) and trienes (*CT*). The *PI* was determined according to the method CD-8b90 proposed by the American Oil Chemists’ Society [50], with some modifications. Briefly, extracted oil from oleogel samples (*n* = 3 for each oleogel batch) was dissolved in chloroform, adding afterward glacial acetic acid, water, potassium iodide, and potato starch. Titration with sodium thiosulfate until the solution turned transparent allowed to calculate the peroxide index in meq O_2_/kg. *CD* and *CT* were determined by the methodology proposed by ISO 3656:2011 [51], with slight modifications. Oil samples (*n* = 3) were weighed (0.01–0.03 g) in a volumetric flask and added to 25 mL of isooctane. The flask was introduced in an ultrasonic bath for 5 min and the resulting solution was poured into quartz cuvettes and measured the absorbance (233 and 268 nm for conjugated dienes and trienes, respectively) in a spectrophotometer (Genesys 10 UV, Thermo Spectronic, Menlo Park, CA, USA).

### 3.8. Color Features of Emulsion and Oleogel

Emulsion and oleogel color (*n* = 3) were measured by a colorimeter (CR-400, Konica Minolta, Osaka, Japan), previously calibrated by a “total blank”. Color evaluation was performed by the CIELAB coordinates (L*, a*, b*). Color was measured before (reference), during, and after the drying process to evaluate the total color difference, Δ*E*, as the moisture decreased by means of Equation (8):

(8) $$∆E=\sqrt{{\left(L^{*}-L_{0}^{*}\right)}^{2}+{\left(a^{*}-a_{0}^{*}\right)}^{2}+{\left(b^{*}-b_{0}^{*}\right)}^{2}}$$

where L*, a*, and b* are the color coordinates of emulsion during drying and subscript 0 corresponds to the color of fresh (time 0) samples.

### 3.9. Statistical Analysis

Statistical analysis was carried out with the software IBM SPSS Statistics 29 (IBM Corp, Armonk, NY, USA). Variance with a confidence level > 95% was determined with the General Lineal Model (*GLM*) method for all dependent variables of the study (texture properties, *OBC*, oxidation, and color). The drying temperature and chitosan concentration were considered as independent variables in the model. 

## 4. Conclusions

Emulsions’ drying kinetics only showed a falling rate period with the highest drying rates at 70 °C. Higher drying temperatures (80 °C) did not shorten significantly the drying times, nor were the oleogel characteristics improved, possibly due to the rapid formation of a superficial crust which hindered proper emulsion drying and subsequent oleogel formation. The Page model was selected from tested empirical models to successfully fit experimental drying kinetics. The effective diffusivity of water varied between 0.75·10^−10^ m^2^/s at 50 °C and 0.8% concentration and 1.58·10^−10^ m^2^/s at 70 °C and 0.7% concentration, allowing satisfactory fittings of experimental drying kinetics. 

The color parameters of oleogels changed with the drying temperature and followed the same trend with chitosan concentration in both air- and freeze-dried samples: L* increasing with temperature at low chitosan content and decreasing at high chitosan content, while a* and b* increased but in a smaller range. Rheological analysis revealed that stable and well-structured oleogels were obtained. Intermediate drying temperatures (60–70 °C) provided the highest elastic moduli, while the weakest structure was obtained in freeze-dried oleogels. The texture results indicated that hardness increased with chitosan content and decreased with drying temperature, while adhesiveness and cohesiveness did not show significant variations among the tested oleogels. Elasticity was higher in oleogels presenting the lowest hardness values. The *OBC* values were higher in air-dried oleogels compared to freeze-dried ones. The highest *OBC* values were achieved at 60 and 70 °C, and 0.8% chitosan concentration. *OBC* values higher than 96% were only obtained with an oleogel with a strength higher than 50,000 Pa.

Concerning the oleogels’ oxidative stability, a significant difference was observed between the air-drying and freeze-drying methods. In the air-drying method, it was found that drying time was crucial for reducing oxidation, as the temperature range was moderately low (50–80 °C). Concerning chitosan (and vanillin) content, no significant improvement in oxidative stability was observed increasing the content of the crosslinkers.

Finally, further research is necessary to corroborate and improve the results obtained, exploring how other oil/water ratios, the use of different chitosan types of marine origin, and the presence of other compounds or emulsion methods influence the characteristics and quality of chitosan-based oleogels. 

## Figures and Tables

**Table 1:** Model parameters (*k*, *a*, and *n*) and statistical results (coefficient of determination (*R^2^*), lumped measure of the goodness (*φ*), and sum of squared errors (*SSE*)) for drying kinetics of tested emulsions ^1^.

Chitosan Concentration (% *w*/*w*)	Drying Temperature (°C)	Model: Newton		*R^2^*	*φ*	*SSE*
		*k* (1/min)				
0.7	50	0.0293 ^d^		0.993	144.89	0.00095
	60	0.0342 ^c^		0.990	129.03	0.00123
	70	0.0566 ^a^		0.996	218.95	0.00037
	80	0.0549 ^b^		0.995	295.21	0.00037
0.8	50	0.0272 ^D^		0.990	90.408	0.00150
	60	0.0304 ^C^		0.986	58.122	0.00160
	70	0.0494 ^A^		0.992	201.98	0.00081
	80	0.0418 ^B^		0.992	171.62	0.00098
**Chitosan Concentration (% *w*/*w*** **)**	**Drying Temperature (°C)**	**Model: Henderson–** **Pabis**		***R^2^***	***φ***	***SSE***
		***k*** **(1/min)**	***a*** **(-)**			
0.7	50	0.0271 ^d^	0.933 ^b^	0.992	186.52	0.00054
	60	0.0315 ^c^	0.927 ^c^	0.989	238.92	0.00079
	70	0.0554 ^a^	0.979 ^a^	0.996	255.73	0.00029
	80	0.0537 ^b^	0.976 ^a^	0.996	307.76	0.00032
0.8	50	0.0243 ^D^	0.913 ^B^	0.989	155.58	0.00084
	60	0.0267 ^C^	0.898 ^C^	0.985	96.414	0.00094
	70	0.0468 ^A^	0.950 ^A^	0.991	304.93	0.00062
	80	0.0391 ^B^	0.938 ^A^	0.991	269.77	0.00074
**Chitosan Concentration (% *w*/*w*** **)**	**Drying Temperature (°C)**	**Model: Page**		***R^2^***	***φ***	***SSE***
		***k*** **(1/min^n^)**	***n*** **(-)**			
0.7	50	0.0557 ^c^	0.824 ^c^	0.998	200.14	0.00012
	60	0.0717 ^b^	0.791 ^d^	0.998	348.08	0.00012
	70	0.0745 ^a^	0.910 ^a^	0.998	272.14	0.00018
	80	0.0746 ^a^	0.903 ^b^	0.998	279.01	0.00017
0.8	50	0.0637 ^C^	0.768 ^C^	0.998	780.87	0.00009
	60	0.0776 ^B^	0.741 ^D^	0.997	232.56	0.00019
	70	0.0857 ^A^	0.831 ^A^	0.997	433.27	0.00019
	80	0.0793 ^A^	0.811 ^B^	0.998	440.80	0.00011

^1^ Standard deviations of *k* values are ±0.0002 and for a and n values ±0.001. Different superscripts (a, b, c, capital letters for 0.8% *w*/*w* chitosan content) indicate significant differences (*p* < 0.05) among mean values at different temperatures according to Duncan’s test.

**Table 2:** Water effective diffusion coefficient during drying for tested emulsions and statistical data to evaluate fitting goodness ^1^.

Chitosan Concentration (% *w*/*w*)	Drying Temperature (°C)	$D_{\mathrm{eff}}\cdot$10^−10^ (m^2^/s)	*R^2^*	*φ*	*SSE*
0.7	50	0.81 ^c^	0.989	1438.48	0.00032
	60	0.95 ^b^	0.995	1389.85	0.00058
	70	1.58 ^a^	0.995	5579.02	0.00015
	80	1.53 ^a^	0.995	2727.10	0.00032
0.8	50	0.75 ^D^	0.987	1010.44	0.00059
	60	0.84 ^C^	0.992	993.935	0.00079
	70	1.36 ^A^	0.993	1906.65	0.00049
	80	1.15 ^B^	0.997	2012.56	0.00041

^1^ Standard deviations of *D_eff_* values are ±0.02. Different superscripts (a, b, c, capital letters for 0.8% *w*/*w* chitosan content) indicate significant differences (*p* < 0.05) among mean values at different temperatures according to Duncan’s test.

**Table 3:** Color coordinates for tested dried solids and oleogels ^1^.

Sample		Oleogel			Dried Solid		
C (% *w*/*w*)	*T* (°C)	L*	a*	b*	L*	a*	b*
0.7	50	19.60 ± 0.52 ^b^	2.25 ± 0.05 ^a^	12.35 ± 0.98 ^b^	51.56 ± 1.08 ^b^	−2.38 ± 0.28 ^b^	21.19 ± 1.05 ^b^
	60	19.04 ± 3.20 ^b^	1.24 ± 0.83 ^a^	13.26 ± 0.47 ^b^	50.85 ± 1.59 ^b^	−1.81 ± 0.17 ^b^	20.33 ± 0.64 ^b^
	70	22.35 ± 0.81 ^b^	1.86 ± 0.01 ^a^	14.57 ± 0.42 ^b^	49.03 ± 0.43 ^b^	−0.23 ± 0.42 ^a^	23.07 ± 0.54 ^b^
	80	24.18 ± 1.58 ^ab^	0.98 ± 0.02 ^a^	15.12 ± 1.41 ^b^	52.25 ± 1.91 ^b^	−1.69 ± 0.13 ^b^	22.14 ± 0.77 ^b^
	FD	29.94 ± 0.39 ^a^	−1.84 ± 0.25 ^b^	19.40 ± 0.90 ^a^	58.34 ± 0.29 ^a^	−5.62 ± 0.12 ^c^	28.77 ± 0.66 ^a^
0.8	50	29.43 ± 1.00 ^A^	−0.33 ± 0.15 ^D^	16.97 ± 0.53 ^AB^	49.27 ± 0.21 ^B^	−1.99 ± 0.47 ^A^	18.43 ± 0.17 ^B^
	60	24.72 ± 0.59 ^B^	0.42 ± 0.14 ^C^	15.24 ± 0.49 ^B^	49.27 ± 0.28 ^B^	−1.44 ± 0.59 ^A^	20.60 ± 0.89 ^AB^
	70	23.08 ± 1.48 ^B^	2.16 ± 0.04 ^A^	14.81 ± 1.06 ^B^	52.52 ± 2.91 ^AB^	−0.92 ± 0.87 ^A^	22.79 ± 2.15 ^A^
	80	25.87 ± 0.07 ^B^	1.05 ± 0.09 ^B^	17.21 ± 0.46 ^AB^	51.01 ± 2.07 ^AB^	−1.04 ± 0.11 ^A^	22.53 ± 0.06 ^A^
	FD	29.51 ± 0.25 ^A^	−1.92 ± 0.06 ^E^	18.85 ± 0.43 ^A^	56.02 ± 0.72 ^A^	−4.37 ± 0.06 ^B^	24.51 ± 0.25 ^A^

^1^ Different superscripts (a, b, c, capital letters for 0.8% *w*/*w* chitosan content) indicate significant differences (*p* < 0.05) among mean values at different temperatures according to Duncan’s test.

**Table 4:** Values of elastic value, *G’* (Pa), at the frequency of 1 Hz of tested oleogels ^1^.

Chitosan Concentration (% *w*/*w*)	Drying Temperature (°C)	*G*′ at 1 Hz (Pa)
0.7	50	36,750 ± 3100 ^b^
	60	80,800 ± 4200 ^a^
	70	72,500 ± 500 ^a^
	80	43,100 ± 800 ^b^
	FD	27,360 ± 1500 ^c^
0.8	50	52,500 ±1800 ^B^
	60	59,900 ± 1300 ^A^
	70	54,700 ± 1400 ^B^
	80	44,000 ± 1700 ^C^
	FD	45,700 ± 3200 ^BC^

^1^ Different superscripts (a, b, c, capital letters for 0.8% *w*/*w* chitosan content) indicate significant differences (*p* < 0.05) among mean values at different temperatures according to Duncan’s test.

**Table 5:** Oil binding capacity (*OBC*) of tested oleogels ^1^.

Chitosan Concentration (% *w*/*w*)	Drying Temperature (°C)	*OBC* (%)
0.7	50	94.35 ± 2.90 a
	60	97.39 ± 1.44 a
	70	98.90 ± 0.56 a
	80	93.59 ± 2.34 a
	FD	91.79 ± 1.56 a
0.8	50	96.97 ± 1.91 AB
	60	98.13 ± 0.55 A
	70	98.80 ± 0.06 A
	80	94.50 ± 0.69 AB
	FD	93.03 ± 1.78 B

^1^ Different superscripts (a, b, c, capital letters for 0.8% *w*/*w* chitosan content) indicate significant differences (*p* < 0.05) among mean values at different temperatures according to Duncan’s test.

## Data Availability

Data will be made available on request.

## Supplementary Materials

The following supporting information can be downloaded at: https://www.mdpi.com/article/10.3390/md22070318/s1, Figure S1: Drying kinetics (main plot) and specific drying rates (subplot) at different air temperatures (°C): (50 ■, 60 ♦, 70 ▲, 80 ●) for the 0.7% *w*/*w* chitosan emulsions. Lines corresponded to the Page model prediction (main plot) and the diffusional model (subplot); Figure S2: Logarithmic relationship thickness vs. moisture for the system 0.7% chitosan and 70 °C; Figure S3: Color coordinates trend with drying time at different air temperatures (°C): (50 ■, 60 ♦, 70 ▲, 80 ●) for the 0.8% *w*/*w* chitosan emulsions (clearer colors for 0.7% *w*/*w*). (a), (b) Brightness coordinate (*L**); (c), (d) red–green coordinate (*a**); (e), (f) yellow–blue coordinate (*b**). Figure S4: Freeze-dried sample with 0.7% chitosan after 48 h storage; Figure S5: Viscous–elastic moduli ratio (*G*″/*G*′ = damping factor) with frequency of tested oleogels at different air temperatures (°C): (50 ■, 60 ♦, 70 ▲, 80 ●) and freeze-dried (●) for the 0.8% *w*/*w* chitosan emulsions (clearer colors for 0.7% *w*/*w*).; Table S1: Initial values of color coordinates (*L**, *a**, *b**) for all the tested systems. FD (freeze-drying).
