# Supplementary material for: Chitosan-Based Oleogels: Emulsion Drying Kinetics and Physical, Rheological, and Textural Characteristics of Olive Oil Oleogels
Source: Mar Drugs. 2024 Jul 17;22(7):318. doi: 10.3390/md22070318 (PMC11278251; doi:10.3390/md22070318)
Supplement: Supplementary file 1 [file marinedrugs-22-00318-s001.zip › marinedrugs-3081045-supplementary.pdf]

Supplementary Material

Chitosan-Based Oleogels: Emulsion Drying Kinetics and Physical, Rheological, and Textural Characteristics of Olive Oil Oleogels

Authors: M. Lama, L. Montes, D. Franco, A. Franco-Uría, R. Moreira

1. Drying kinetics of 0.7% w/w chitosan emulsions

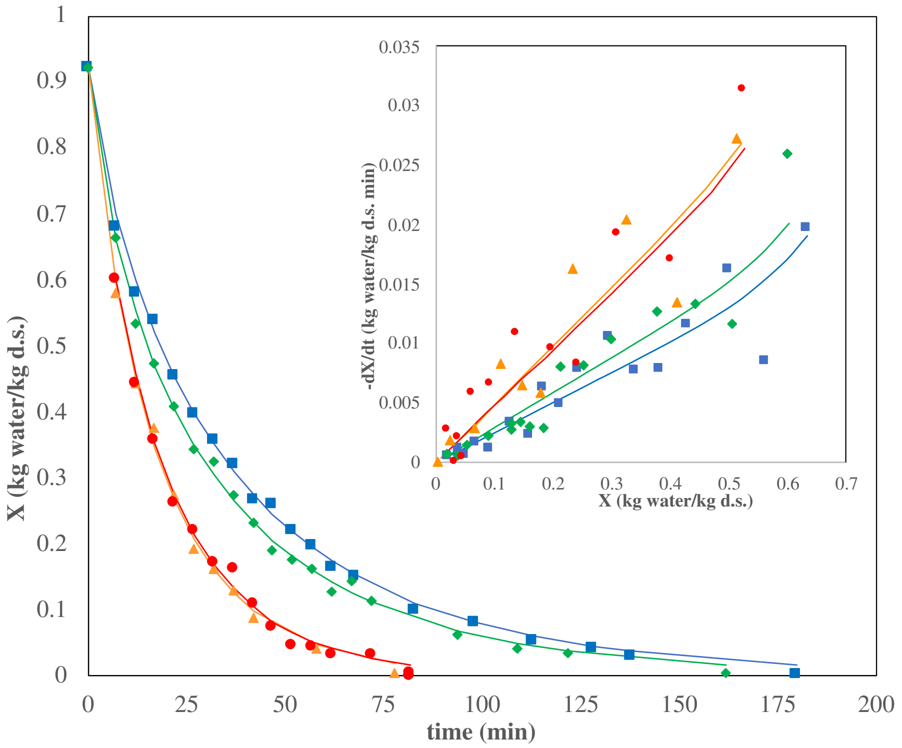

Figure S1. Drying kinetics (main plot) and specific drying rates (subplot) at different air temperatures (ºC): (50 ■,60 ♦,70 ▲,80 ●) for the 0.7% w/w chitosan emulsions. Lines corresponded to the Page model prediction (main plot) and the diffusional model (subplot)

2. Thickness variation during drying

In Figure S.2, the shrinking of a system can be determined by a logarithmic relationship between thickness and moisture.

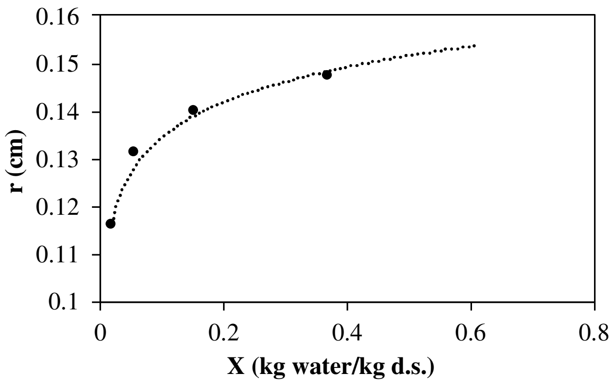

Figure S2. Logarithmic relationship thickness vs moisture for the system 0.7% chitosan and 70ºC

This relationship is defined by Eq. S.1:

$$r(X) = 0.0107\ln(X) + 0.159$$

Where r is the thickness in m and X is the absolute moisture (kg water/kg d.s.). Fitting present an R^2= 0.979 and a RMSE of 0.00575.

3. Color

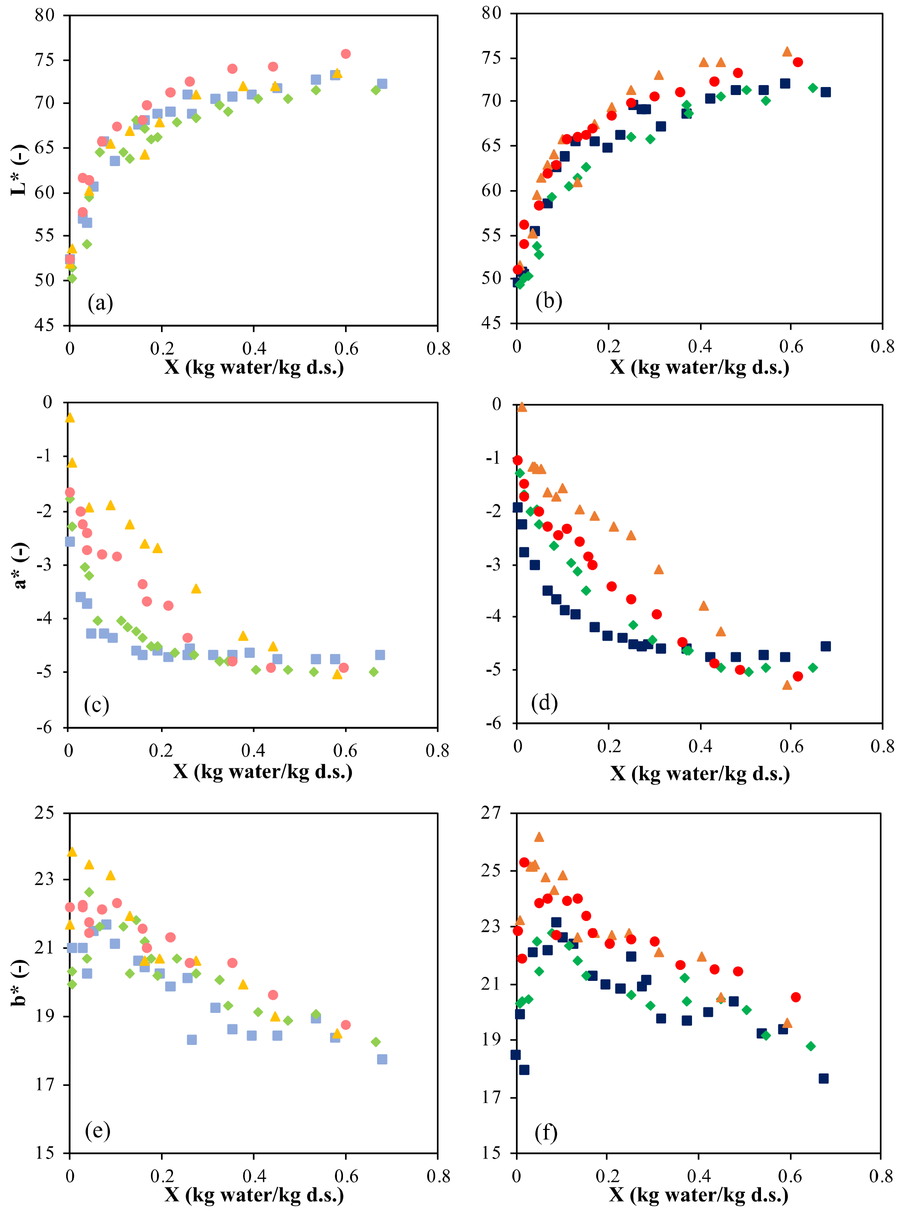

Figure S3: Color coordinates trend with drying time at different air temperatures (ºC): (50 ■,60 ♦,70 ▲,80 ●) for the 0.8% w/w chitosan emulsions (clearer colors for 0.7% w/w). (a), (b) Brightness coordinate (L*); (c), (d) red-green coordinate (a*); (e), (f) yellow-blue coordinate (b*).

Table S1. Initial values of color coordinates (L*, a*, b*) for all the tested systems. FD (Freeze-drying)

Coordinate	L*	a*	b*
Sample			
50°C – 0.7%	72.38 ± 0.94	-4.26 ± 0.08	16.58 ± 0.82
50°C – 0.8%	72.78 ± 1.27	-4.11 ± 0.09	16.57 ± 0.69
60°C – 0.7%	71.12 ± 1.05	-4.36 ± 0.10	15.74 ± 0.87
60°C – 0.8%	73.44 ± 0.98	-4.48 ± 0.05	17.15 ± 0.56
70°C – 0.7%	74.15 ± 2.74	-4.50 ± 0.07	16.17 ± 0.44
70°C – 0.8%	77.99 ± 1.21	-4.75 ± 0.09	16.97 ± 0.70
80°C – 0.7%	76.68 ± 1.13	-4.43 ± 0.08	17.12 ± 0.67
80°C – 0.8%	76.20 ± 0.53	-4.66 ± 0.04	19.18 ± 0.36
FD – 0.7%	73.28 ± 1.03	-4.35 ± 1.03	17.26 ± 0.52
FD – 0.8%	76.68 ± 1.11	-4.43 ± 1.11	17.12 ± 0.68

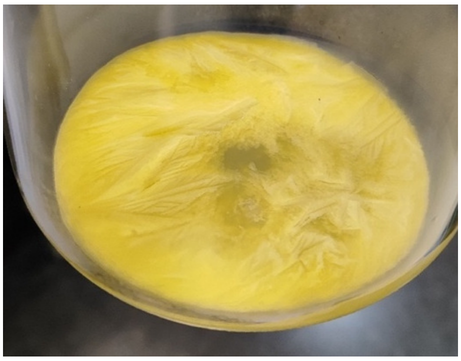

Figure S4. Freeze-dried sample with 0.7% chitosan after 48h storage.

4. Rheological properties

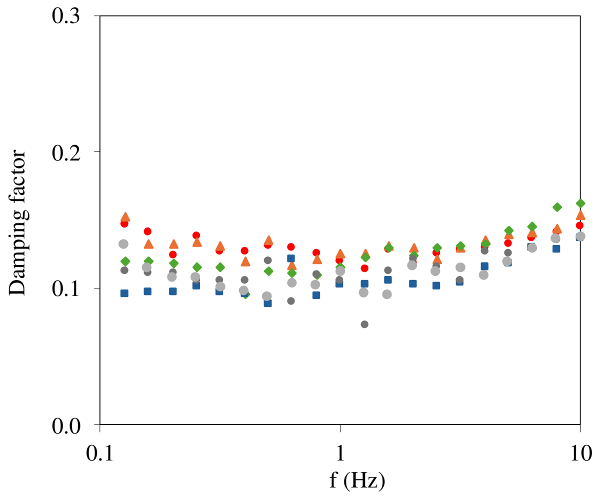

Figure S5. Viscous-elastic moduli ratio (G´´/G´ = damping factor) with frequency of tested oleogels at different air temperatures (ºC): (50 ■,60 ♦,70 ▲,80 ●) and freeze-dried (●) for the 0.8% w/w chitosan emulsions (clearer colors for 0.7% w/w).
